# An interpretable machine learning tool for in-home monitoring of agitation episodes in people living with dementia: a proof-of-concept study

**DOI:** 10.1016/j.eclinm.2024.103032

**Published:** 2025-01-20

**Authors:** Marirena Bafaloukou, Ann-Kathrin Schalkamp, Nan Fletcher-Lloyd, Alex Capstick, Chloe Walsh, Cynthia Sandor, Samaneh Kouchaki, CR&T Group, Ramin Nilforooshan, Payam Barnaghi

**Affiliations:** a Department of Brain Sciences, Imperial College London, UK; b UK Dementia Research Institute at Care Research and Technology Centre, UK; c UK Dementia Research Institute at Imperial College London, UK; d Surrey and Borders Partnership NHS Foundation Trust, Leatherhead, UK; e University of Surrey, UK; f Great Ormond Street Hospital NHS Foundation Trust, London, UK

**Keywords:** Dementia care, Agitation, Machine learning, Remote monitoring, Digital health tools

## Abstract

**Background:**

Agitation affects around 30% of people living with dementia (PLwD), increasing carer burden and straining care services. Agitation identification typically relies on subjective clinical scales and direct patient observation, which are resource-intensive and challenging to incorporate into routine care. Clinical applicability of data-driven methods for agitation monitoring is limited by constraints such as short observational periods, data granularity, and lack of interpretability and generalisation. Current interventions for agitation are primarily medication-based, which may lead to severe side effects and lack personalisation. Understanding how real-world factors interact with agitation within home settings offers a promising avenue towards identifying potential personalised non-pharmacological interventions.

**Methods:**

We used longitudinal data (32,896 person-days from n = 63 PLwD) collected using in-home monitoring devices between December 2020 and March 2023. Employing machine learning techniques, we developed a monitoring tool to identify the presence of agitation during the week. We incorporated a traffic-light system to stratify agitation probability estimates supporting clinical decision-making, and employed the SHapley Additive exPlanations (SHAP) framework to enhance interpretability. We designed an interactive tool that enables the exploration of personalised non-pharmacological interventions, such as modifying ambient light and temperature.

**Findings:**

Light Gradient-boosting Machine (LightGBM) achieved the highest performance in identifying agitation over an 8-day period with a sensitivity of 71.32% ± 7.38 and specificity of 75.28% ± 7.38. Implementing the traffic-light system for stratification increased specificity to 90.3% ± 7.55 and improved all metrics. Key features for identifying agitation included low nocturnal respiratory rate, heightened alertness during sleep, and increased indoor illuminance, as revealed by statistical and feature importance analysis. Using our interactive tool, we identified indoor lighting and temperature adjustments as the most promising and feasible intervention options within our cohort.

**Interpretation:**

Our interpretable framework for agitation monitoring, developed using data from a dementia care study, showcases significant clinical value. The accompanying interactive interface allows for the *in-silico* simulation of non-pharmacological interventions, facilitating the design of personalised interventions that can improve in-home dementia care.

**Funding:**

This study is funded by the 10.13039/501100017510UK Dementia Research Institute [award number UK DRI-7002] through UK DRI Ltd, principally funded by the 10.13039/501100000265Medical Research Council (MRC), and the 10.13039/501100000266UKRI Engineering and Physical Sciences Research Council (EPSRC) PROTECT Project (grant number: EP/W031892/1). Infrastructure support for this research was provided by the 10.13039/501100013342NIHR Imperial Biomedical Research Centre (BRC) and the UKRI Medical Research Council (MRC). P.B. is also funded by the Great Ormond Street Hospital and the 10.13039/501100000287Royal Academy of Engineering. C.S. is supported by the 10.13039/501100017510UK Dementia Research Institute [award number UK DRI-5209], a UKRI Future Leaders Fellowship [MR/MR/X032892/1] and the Edmond J. Safra Foundation. R.N. is funded by 10.13039/501100017510UK Dementia Research Institute [award number UK DRI-7002] and the 10.13039/501100000266UKRI Engineering and Physical Sciences Research Council (EPSRC) PROTECT Project (grant number: EP/W031892/1). M.B. and A.K.S. are funded by the 10.13039/501100017510UK Dementia Research Institute [award number UKDRI-7002 and UKDRI-5209]. N.F.L., A.C., C.W. and S.K. are funded by the 10.13039/501100017510UK Dementia Research Institute [award number UK DRI-7002].

**Research in context**

**Evidence before this study**

A search in PubMed using the keywords agitation, dementia, machine learning, and sensor yielded only four studies, and one review article published between 2018 and 2024. These studies refer to using machine learning (ML) models to detect agitation onset through multimodal sensor data. However, they are limited by small participant numbers, challenges in scalability within real-world longitudinal settings, and narrow time windows, which can increase the likelihood of false alerts and reduce interpretability. Moreover, none of these studies focuses on monitoring episodes of agitation over a week in a way that could substitute existing clinical scales for people living with dementia at home. This underscores the need for scalable, interpretable, and clinically applicable solutions to improve the management of agitation in dementia care.

**Added value of this study**

Our study utilised a large dataset from an in-home monitoring study with extensive participant and person-day coverage to examine agitation episodes in people living with dementia (PLwD). We introduced an ML model with enhanced explainability, identifying key factors for agitation. We implemented a traffic-light system, which categorises agitation probability estimates into different credence levels, improving clinical management by reducing false alerts and focusing on high-confidence cases. We present the first interactive tool for agitation monitoring that also allows *in-silico* testing of non-pharmacological interventions, enabling personalised treatment strategies to be refined before real-world application. This is the first study that focuses on introducing a weekly monitoring tool for agitation in PLwD.

**Implications of all the available evidence**

The proposed agitation monitoring and analysis tool introduces a novel approach and a valuable clinical decision support system for managing agitation in PLwD. The insights generated from the ML model provide opportunities for designing new clinical trials focused on non-pharmacological interventions for agitation in PLwD. Additionally, the study highlights the relationship of ambient temperature, light, and sleep with agitation, offering new perspectives on these complex interactions.

## Introduction

Agitation is a neuropsychiatric symptom that affects more than 30% of people living with dementia (PLwD),^1^ and is characterised by a 93% recurrence rate.^2^ Agitation in individuals with cognitive impairment or dementia is defined as persistent behaviours that indicate emotional distress, can lead to significant disability, and are not solely attributable to another disorder.^3^ Agitation manifests as excessive motor activity and verbal or physical aggression. However, agitation is not synonymous with aggression; it is also characterised by non-aggressive actions, such as wandering and repetition.^3^, ^4^, ^5^ Such episodes are associated with adverse healthcare events, including fall-related injuries.^6^ Current clinical practice for managing agitation in PLwD primarily relies on pharmaceutical interventions, such as prescribing antipsychotic drugs. However, these medications may cause severe side effects, including pneumonia, acute kidney injury, venous thromboembolism, and stroke.^7^ Agitation could also affect the emotional and mental well-being of carers.^8^ Identification and appropriate treatment of agitation are essential for mitigating associated risks, lowering healthcare expenses, and alleviating carer strain.^6^

Various clinical measures have been developed to assess agitation, which can be grouped into two main categories: informant ratings, including the Cohen-Mansfield Agitation Inventory (CMA-I),^4^ and direct observational assessments such as the Pittsburgh Agitation Scale.^9^ Informant ratings, as noted by Cohen-Mansfield et al., can be unreliable due to the personal bias of the informant, memory inaccuracies, and stress. Direct observational assessments are more objective; however, they require trained staff to monitor behaviour, making these assessments resource-intensive and not applicable to at-home settings.^10^^,^^11^

Applying machine learning (ML) methods to continuously collected remote monitoring data can address the above mentioned limitations.^12^, ^13^, ^14^, ^15^ Spacojevic et al. proposed a Random Forest (RF) classifier to detect agitation using feature-engineered motion and physiological sensor data in 1-min windows, collected from 20 participants over a 600-day period. However, the small number of participants in their dataset limits the generalisability of their findings. Moreover, the small data granularity (1-min) restricts the exploration of long-term agitation-driving factors, which are crucial for effectively managing agitation episodes in real-world settings. Similarly, as discussed by Deters et al. in their recent review, smaller windows may impede the capture of broader behavioural patterns essential for identifying agitation episodes.^15^ Khan et al. applied deep sequential modelling on the same dataset, achieving similar performance to the RF but with reduced interpretability due to the complexity of deep learning (DL), further constraining the model's clinical applicability.^12^ In our previous work, we integrated activity and physiological data–from 46 PLwD, collected over 2 years–into a DL model to predict agitation using a 6-h window.^14^ The model's high false positive rate and the complexity of the DL model, providing limited insights into the underlying factors, hindered its clinical utility. Hekmatiathar et al., incorporated interior ambient data collected over 64 days, from the living environment of one participant in a DL model, to forecast agitation using a rolling window of 30 min.^16^ Their approach relied on frequent validation, thereby increasing the likelihood of false positives, which could disrupt patients' and carers' routines. Moreover, their reliance solely on environmental data and validation on only one participant limit the model's clinical reliability.

Overall, existing studies on the identification of agitation lack generalisability due to small data sizes and limited interpretability, while studies on agitation prediction lack clinical and care applicability, as agitation antecedents are often unpredictable.^17^ This highlights the need to shift towards agitation monitoring and develop transparent and interpretable ML models that encompass a diverse range of variables and can capture broader agitation patterns.

The National Institute of Health and Care Excellence (NICE) in the UK recommends prioritising non-pharmacological interventions, such as sensory stimulation, as the primary strategy to address dementia-related agitation.^18^ This is driven by concerns surrounding potential side effects linked to medications and their limited long-term efficacy.^19^ Light exposure^20^ and music-related interventions have shown promise for the treatment of agitation in dementia.^21^ However, existing studies for non-pharmacological interventions are limited by small sample sizes and inadequate reporting of findings, preventing a comprehensive understanding of non-pharmacological interventions’ efficacy.^21^ Optimal design of such interventions requires a personalised study of individual patterns, symptoms and preferences^22^, an area which remains under-explored.

Here, we used real-world data from an observational clinical study called Minder^23^ to develop an ML framework that identifies the occurrence of agitation episodes within a week. Using the passive monitoring data from Minder, we integrated a diverse array of features, including sleep measures, activity levels, and environmental parameters, into our model. Our dataset comprised data collected over 32,896 person-days from 63 PLwD, with a total of 512 weeks labelled for agitation occurrence. Through the assessment of feature importance, we sought to provide information on the model's decision-making process. We developed a novel interactive tool, tailored for designing and validating non-pharmacological interventions, based on real-world insights from the weekly personalised agitation monitoring.

## Methods

### Study design and population

The Minder study was initiated in collaboration with Imperial College London, the University of Surrey and Surrey and Borders Partnership NHS Trust.^23^ Eligible study participants included adults older than 50 years with a clinically ascertained diagnosis of dementia or mild cognitive impairment and current or previous treatment at a psychiatric unit. In total, 127 participants have been recruited (Table 1). Most participants live with carers or study partners who attend clinical assessments with them. More details on the study design are available in subsubsection S1.4.1.

**Table 1:** Demographics.

Characteristic	Entire minder cohort	Agitation cohort	Statistical analysis cohort
**Total**	127	63	29
**Diagnosis**			
Alzheimer’s disease	59	36	14
Vascular dementia	7	5	3
Frontotemporal dementia	6	1	1
Parkinson’s disease	4	3	2
Unspecified demantia	31	13	7
Other and mixed	20	5	2
**Age group**			
50–60	1	0	0
60–70	12	7	1
70–80	29	13	7
80–90	55	31	18
90–100	27	12	3
N/A	3	0	0
**Gender**			
Male	69	41	23
Female	57	22	6
Unspecified	1	0	0
**Ethnicity**			
White	100	52	23
Asian/Asian British	8	4	2
Black/African/Caribbean/Black	3	1	0
Mixed/Multiple ethnic groups	1	0	0
N/A	15	4	4
**Household**			
Multiple occupancy	71	49	25
Single occupancy	49	13	3
N/A	7	1	1

Demographics of participants in the entire minder cohort (n = 127), participants that were used in the agitation analysis (n = 63) and participants used in the statistical analysis (n = 29) are shown. The number of participants in each group is displayed.

### Data collection

Demographic data were collected during the baseline assessment. In-home monitoring data was continuously collected using low-cost sensors placed in the participants' homes. These devices include passive infrared sensors (PIR), sleep mats, and door and kitchen appliance sensors. Here, we included data from the PIR sensors comprising of activity, indoor light, and indoor temperature. We also integrated data from the sleep mat, which is placed underneath the participant's side of the mattress and monitors respiratory and heart rates, and nighttime events. Additionally, we included outdoor light, outdoor temperature, and weather data sourced from the Visual Crossing Weather API https://www.visualcrossing.com,^24^ specifically for the Surrey area where most enrolled participants resided. Lux illuminance units for outdoor light were derived from solar irradiance values, directly available on Visual Crossing, using the conversion factor of 122.^25^ For a layout of all sensors used refer to Figure S1.

Clinical psychometric and cognitive assessment tools, including the Neuropsychiatric Inventory (NPI), were administered within the Minder study, to gather behavioural and cognitive data during regular (3-month) visits. Additionally, a weekly behavioural monitoring questionnaire process was implemented. During this weekly monitoring process, study partners and/or participants were contacted to report the presence or absence of participants’ behavioural symptoms regarding the preceding week. This included symptoms such as agitation, delusions, hallucinations, depression, and anxiety.

### Data labelling

In our study, agitation status was determined in a two-stage verification method, through the responses from the weekly monitoring process. Trained research staff, having completed both NIHR Good Clinical Practice (GCP) and Valid Informed Consent training, compiled unstructured notes based on their weekly interactions with carers/participants and labelled the weeks for each participant as presence or absence of agitation. Agitation presence was indicated if participants exhibited increased restlessness, irritability, resistance to care, or episodes of verbal or physical aggression. Absence of agitation was marked by the lack of these symptoms across the week and observation of the participant's usual behaviour patterns. These criteria were developed based on standard agitation assessment tools and definitions of agitation.^3^^,^^26^ We conducted a review to confirm the correspondence of the notes with the labels (Table S1). We only retained verified instances of absence of agitation, supported by notes, to avoid including false negatives from cases where participants or carers were unable to report on the previous week. This approach helped ensure consistency and reproducibility in our labelling process. Examples from the notes can be found in Table S1.

The whole preceding week, including the day of the reporting itself (8 days), was labelled as a binary target variable representing the presence or absence of agitation. An 8-day rolling window was applied to consistently extract 8 days of data preceding an event even if the time between events was smaller than 8. This approach was chosen to accurately simulate the model's operation in real-world use. The weekly nature of the labels allowed for the observation of the period surrounding agitation episodes, facilitating the identification of broader agitation patterns and factors related to agitation. The weekly monitoring responses were chosen over the more detailed NPI ones, due to the weekly granularity enabling us to use the labels in association with passively collected sensor data. Using weekly labels further has the potential to reduce participant burden by minimising the frequency of contact for agitation events' verification and questionnaire administration.

**Fig. 1:**
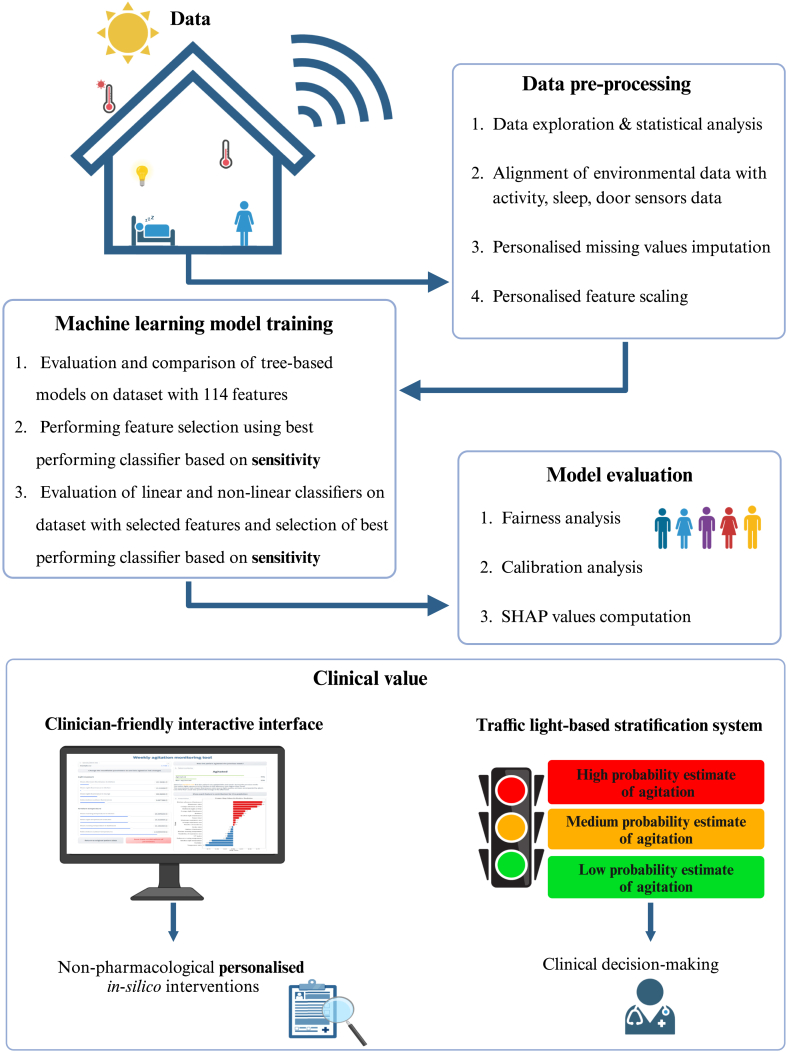
**Overview of approach**. The data processing and analysis pipeline are shown, illustrating the specific steps followed. Our two strategies for enhanced clinical value are showcased: an interactive interface for personalised in-silico intervention experiments and a traffic-light system to minimise false alerts. Figure was created with Biorender.

For our experimentation (Fig. 1), we used the labelled data collected in the Minder study, from 15/07/2021 to 16/03/2023. To develop the ML model, we included individuals for whom the presence or absence of agitation episodes was recorded, leading to a final sub-cohort of 63 (41 male and 22 female participants) with a total of 242 positive and 270 negative agitation-labelled weeks. Most of those participants resided with carers (49 out of 63). The demographics of the agitation sub-cohort are provided in Table 1.

### Data exploration and pre-processing

To determine which source of light and temperature (indoor and outdoor) data to include at different times during the day, we used PIR, door sensors, and the sleep mat to determine the location of the subject. Only indoor light and temperature values that corresponded to simultaneous (resolution = 1 h) activity data from PIR sensors were retained. Outdoor values (daily averages) were retained only for days when there was an indication that the participant was not indoors. This was confirmed by only retaining days for which there was use of the front or back door and otherwise inactivity in the house. Indoor light, indoor temperature, and activity data were aggregated over time windows of 6 h, representing different periods of the day: morning, afternoon, evening, and night, to capture fluctuations in environmental variables and activity signatures in the house throughout the day. These 6-h windows were then averaged weekly. For sleep features collected from the bed mats, we used daily aggregated data and we subsequently aggregated weekly. In addition to weekly sleep feature averages, we calculated standard deviations (SD) of each sleep feature over the week to create additional informative metrics representing the variability in sleep patterns associated with agitation events. In our approach, we chose to engineer weekly features from our sensor data to effectively manage our weekly agitation labels. By aggregating data on a weekly basis, we minimise the noise and variability that can result from daily fluctuations. This approach allows us to establish reliable and stable weekly averages, which are less affected by the randomness of daily occurrences (e.g., presence of visitors). For a comprehensive overview of our data sources and aggregation strategies, refer to the framework in Figure S2.

In our exploratory analysis, after assessing normality using Shapiro–Wilk test, we conducted two-sided paired Student's T-tests or Wilcoxon-signed rank tests to compare various extracted measures describing sleep, illuminance and ambient temperature between agitation and non-agitation weeks. For this, we only included participants who had both positive and negative agitation events (n = 29) (Table 1).

For data imputation, once the weekly values for each feature were aggregated and if there was no data for a particular feature for that week, we imputed the missing values. The missing data were considered to be missing at random, as it was typically attributed to passive sensor device outages or server failures, which, due to the passive nature of data collection, could not have been influenced by agitation status (target variable). The data imputation process is described below. We handled each data modality separately during imputation. Missing indoor light, indoor temperature, sleep, and activity labelled data were imputed with the k-nearest neighbours algorithm (*k* = 5) separately for each participant. For missing values that remained, iterative imputation was performed using Bayesian Linear Ridge regression and initial strategy the median, with 10 iterations on unlabelled data from all participants. For the outdoor light and temperature data, when there was no outdoor activity for the whole week, we used the average outdoor values from the entire week. For the imputed activity, light, and temperature data, we mapped the room names to the most common location terms, resulting in Lounge, Kitchen, Bathroom, and Bedroom. As a result of our imputation strategy, we retained only those events for which data across all modalities were available following imputation. In Table S7 we evaluate the final model's sensitivity to different imputation strategies and find that the model is robust to these approaches.

Z-score normalisation was performed to scale each participant's data according to their individual baseline. This involved computing the individual baseline mean and standard deviation (SD), by considering both labelled and unlabelled data (32,896 person-days) collected from each participant throughout their entire participation in the Minder study between December 2020 and March 2023. This scaling procedure was undertaken to mitigate potential individual differences among participants and ensure a standardised and fair comparison across the dataset. Additionally, by normalising each participant's data to their individual baseline, we aimed to account for the influence of carers who lived with the participants. Given that carers were generally healthy individuals with stable routines, we assumed their behaviours remained consistent over time. By normalising each household's weekly signatures against its own baseline, we aimed to mitigate the carer contributions to the sensor data, allowing for a clearer focus on participant-specific changes. 7 participants in total did not have sufficient weekly data to compute reliable statistical properties for specific combinations of time-period and room features (e.g., Bathroom night illuminance or Kitchen night temperature). The missing data varied among participants, with each individual exhibiting gaps in different features. This variability stemmed from differences in home signature, behaviour, and duration of study participation among participants. To address this, we substituted the missing average or SD feature value with the average statistical properties derived from other participants (Table S7).

### Feature selection

We derived 114 variables from the sensor data, including sleep, activity, indoor and outdoor light exposure, indoor and outdoor temperature, and seasonality (Table S2). Feature selection was performed to prevent overfitting and enhance model generalisability. We used the SHapley Additive exPlanation (SHAP)^27^ values, computed by a Random Forest Classifier (RF), using 10 folds. All data splits were stratified and were performed with a grouping strategy based on individual participant ID (ID-grouping), preventing data leakage. The top-ranked features were selected to reflect the features that had a higher contribution to the model decision. We derived all the SHAP values for the features and included a cut-off threshold where the SHAP values had a sharp decline in their importance value. The selected features had SHAP values greater than 0.015347. More information on feature selection can be found in section S1.4.2.

### Machine learning models

To identify the presence of agitation over an 8-day period, we compared the performance of RF,^28^ Light Gradient-Boosting Machine (LightGBM)^29^ Extreme Gradient Boosting (XGBoost),^30^ Adaptive Boosting (ADABoost),^31^ Support Vector Machine (SVM),^32^ Logistic Regression (LR)^33^ and a multilayer-perceptron (MLP). Details are provided in subsections S1.2, S1.3.

The hyperparameters for the models were tuned using grid search on stratified 5-Fold train/validation splits, with the model achieving the highest sensitivity on the validation data selected as the most suitable model. Regularisation techniques (L1 and L2) were also utilised in the models whenever possible to manage model complexity and mitigate overfitting (Details on hyperparameters are provided on Table S6). Model performance was evaluated using stratified, ID-grouping 10-Fold train/test splits. The choice of grouping by participant ID was made to mitigate circularity and overfitting and allow us to fairly evaluate our models by preventing multiple events from the same participants from biasing the results.

Sensitivity was prioritised as the primary metric to enhance the model's capability to sensitively identify episodes of agitation, as desired for medical applications. Performance of the 7 models on the 10 test sets, was compared using two-sided paired T-tests (N = 10). The best-performing model (LightGBM) was identified and used for further analysis.

### Probability estimate stratification

To further assist clinical decision-making, a traffic-light-based system was developed, grouping the agitation probability estimates outputted by our classifier–representing the model's confidence for each instance–into three categories based on defined thresholds: Green (low probability estimate), Amber (medium probability estimate), and Red (high probability estimate).

The thresholds were determined via a 10-fold nested stratified cross-validation approach on the LightGBM, incorporating ID-grouping to ensure that participants used to determine the thresholds were distinct from those used for evaluation. The inner 10-fold cross-validation determined thresholds based on validation set performance, while the outer 10-fold cross-validation evaluated model performance on independent test sets post-threshold application.

We optimised the traffic light model to have a balanced inclusion ratio between the Amber, Green and Red categories. By expanding the boundaries of the Amber group, the false alerts would be decreased and sensitivity would be increased. However, a disproportionate allocation of the results to the Amber group would decrease the specificity of the model.

To avoid this, we focused on jointly increasing sensitivity and specificity, by maximising Youden's J index.^34^ To achieve this, we varied the stratification thresholds with a resolution of 10% and computed the sensitivity and specificity of the predictions on the Green (no agitation determined) and Red categories (agitation determined) in the validation sets (Figure S6). To inform the range of the thresholds, we used a source different from the weekly labels used for model development to avoid circularity. Specifically, we used the NPI questionnaire responses, administered to the entire Minder cohort from July 2020 to March 2023, every three months (Table S10, subsection S1.5.4). The rate of Red (Rr) alerts was determined based on the agitation frequency domain reported in the NPI by the carers. The rate of Green alerts (Gr) was defined, due to agitation episodes being less frequent than their absence (see section S1.5.4). The thresholds were filtered based on the following criteria.

• Ensuring that the Rr is:

15% ≤ Rr ≤ 25%

• Ensuring that the Gr is:

Gr ≥ 25%

• Maximising Youden's J index, defined as Sensitivity + Specificity — 1

When considering all thresholds adhering to these criteria on the validation sets, each average threshold was computed, resulting in 10 averages. During model evaluation on the test sets, the threshold from each respective validation set was used.

The average thresholds from all 10 folds were:

• Green: [0.00, 32.25]
• Amber: [32.25, 78.14]
• Red: [78.14, 100.00]

### Interactive interface

We created an interactive interface using the Gradio library.^35^ The developed tool allows uploading patient data as a CSV file to compute weekly agitation probability estimates based on the trained ML model. The corresponding category from the traffic-light system is displayed and feature contributions are shown using the SHAP values. We defined a set of modifiable factors: {Kitchen afternoon and night illuminance, Lounge night illuminance, illuminance ratio (average indoor:average outdoor), Kitchen morning temperature, Kitchen night temperature, Bathroom evening temperature, temperature ratio (average indoor:average outdoor}. These can be adjusted on the interface using sliding bars enabling a simulation of the effect of non-pharmacological interventions in a personalised framework. The user can download their applied modifications to facilitate the design of interventions. For our *in-silico* experimentation within this paper, we utilise the tuned and trained model from the corresponding fold to which the participant examined belongs in the test set. The tool is hosted on huggingface. The online version employs the LightGBM model tuned and trained on all data available from the 63 participants (https://huggingface.co/spaces/marirena/AgitationMonitoring). To ensure data privacy, we include a synthetic data generator in the online version.

### Ethics

The study received ethical approval from the London-Surrey Borders Research Ethics Committee; TIHM 1.5 REC 19/LO/0102. The study is registered with the National Institute for Health and Care Research (NIHR) in the United Kingdom under the Integrated Research Application System (IRAS) registration number 257561. Participants lacking capacity for informed consent were required to have a study partner or carer who had known them for at least 6 months and was able to attend clinical study assessments with them. Participants received an information sheet outlining how their personal data would be used in compliance with GDPR requirements. All participants provided written informed consent. The capacity to consent was assessed according to Good Clinical Practice, as detailed in the Research Governance Framework for Health and Social Care (Department of Health 2005) and the Mental Capacity Act 2005. If a participant lacking capacity expressed willingness to participate, a personal consultee would sign a declaration of consent. If no personal consultee was available, a professional consultee such as a key worker was sought. This process was detailed in the study protocol and approved by the ethics panel.^23^

### Role of the funding source

The funders were not involved in the study design, data collection, data analysis or writing the manuscript.

## Results

### Agitation episodes are accompanied by poor sleep quality and differences in light exposure

Sleep, illuminance and temperature features, derived from the in-home PIR sensors and the sleep mat, alongside outdoor ambient data, highlight differences between agitation and non-agitation weeks, showing their potential to be used for weekly agitation monitoring. During agitation weeks, the average awake ratio was significantly higher compared to non-agitation weeks (0.27 ± 0.67 vs. −0.18 ± 0.40) (*p*−*value* = 2 × 10^−2^), whereas the average minimum respiratory rate was significantly lower (−0.33 ± 0.68 vs. 0.09 ± 0.33) (*p*−*value* = 2 × 10^−2^) (Table S14). Furthermore, indoor illuminance was significantly higher during agitation weeks compared to non-agitation weeks (0.10 ± 0.25 vs. −0.05 ± 0.19) (*p*−*value* = 2 × 10^−2^), while the indoor to outdoor illuminance (illuminance ratio) was significantly lower (−0.26 ± 0.27 vs. 0.18 ± 0.26) (*p*−*value* < 10^−3^) (Tables S16, S21).

This feature analysis showed that sleep patterns and environmental factors differed during agitation weeks, and could be used in an ML model for weekly monitoring of agitation episodes.

### Light Gradient-Boosting Machine classifier can identify agitation using sensor data

Linear and non-linear classifiers were evaluated for the identification of agitation episodes over an 8-day period (see Table S4). The baseline models that were evaluated included SVM, LR, LightGBM, RF, XGBoost, ADABoost, and an MLP. Model evaluation focused on maximising sensitivity, ensuring the model's effectiveness in serving as a sensitive monitoring tool.

The LightGBM yielded significantly higher sensitivity than logistic regression (*p*−*value <* ×10^−3^) and the other examined tree-based models (*p* − *value* < 5 × 10^−3^) (see Table S5). The LightGBM classifier also achieved higher scores in most performance metrics compared to the examined models (see Table S4). The model showed robust results in calibration (Figure S4), reliability (Figure S5), and bias analysis (Table S8, Table S9, Figure S3).

Incorporating a stratification technique via a traffic-light system, which included an additional Amber category (medium probability estimate), increased sensitivity by 5% and specificity by 15% and improved all metrics. The inclusion of the Amber group reduced the false alerts, increasing the model's clinical applicability. Refer to Table 2 for a comparison of metrics before and after the stratification, and see Figure S8 for visual representations of the receiver operating characteristic (ROC) and precision-recall (PR) curves. More details on stratification are provided in section S1.5.4.

**Table 2:** Performance comparison before and after the application of the stratification technique.

Metric	Binary classification	Traffic-light system
Accuracy	71.36 ± 7.21	78.87 ± 6.88
Fl Score	71.12 ± 7.28	76.70 ± 7.52
Precision	71.81 ± 7.94	80.06 ± 7.73
Sensitivity	71.32 ± 7.38	76.36 ± 7.45
Specificity	75.28 ± 10.43	90.33 ± 7.55
PR AUC	78.60 ± 17.57	81.42 ± 9.99
ROC AUC	77.63 ± 6.59	80.73 ± 9.37

The traffic-light system optimised the thresholds for decision-making, creating Green (low agitation probability estimate), Amber (medium agitation probability estimate), and Red (high agitation probability estimate) categories. The performance metrics from the 10-Fold cross-validation are reported as mean ± standard deviation. Metrics include accuracy, F1-score, precision, sensitivity, specificity, PR AUC (area under the precision-recall curve), ROC AUC (area under the receiver operating characteristic curve).

### Low respiratory rate and high indoor artificial illuminance are key factors in the model's decisionmaking towards agitation episodes

To increase the clinical value of the presented model, we investigated feature importance via SHAP values.

**Fig. 2:**
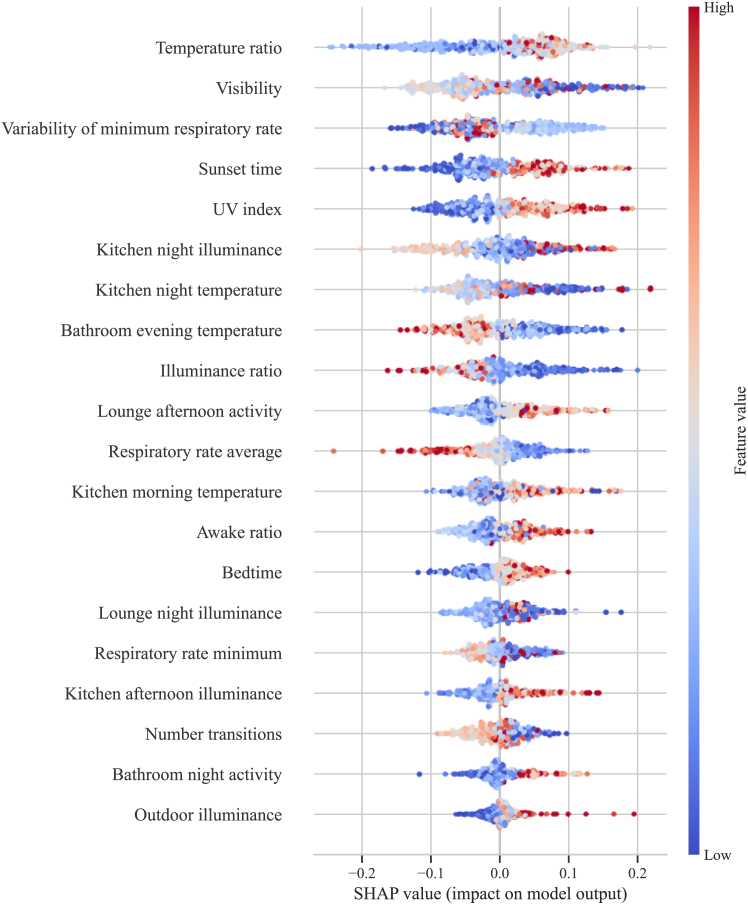
**Model interpretability through feature importance analysis.** The feature importance calculated using SHapley Additive exPlanations (SHAP) on the test sets from the 10-Fold cross-validation is shown in a summary plot. The colour represents the scaled feature value (red corresponding to higher values, blue to lower). The position of the x-axis represents the contribution of each normalised feature value to the positive prediction of agitation.

Fig. 2 demonstrates the contribution of each feature's high (red) and low (blue) values to the model's decision. The most important features for agitation identification were low respiratory rate, high awake ratio, extreme values of visibility and indoor illuminance (both high and low), low illuminance ratio, high temperature ratio and low indoor temperatures.

### Interactive online tool allows simulation of personalised non-pharmacological interventions

**Fig. 3:**
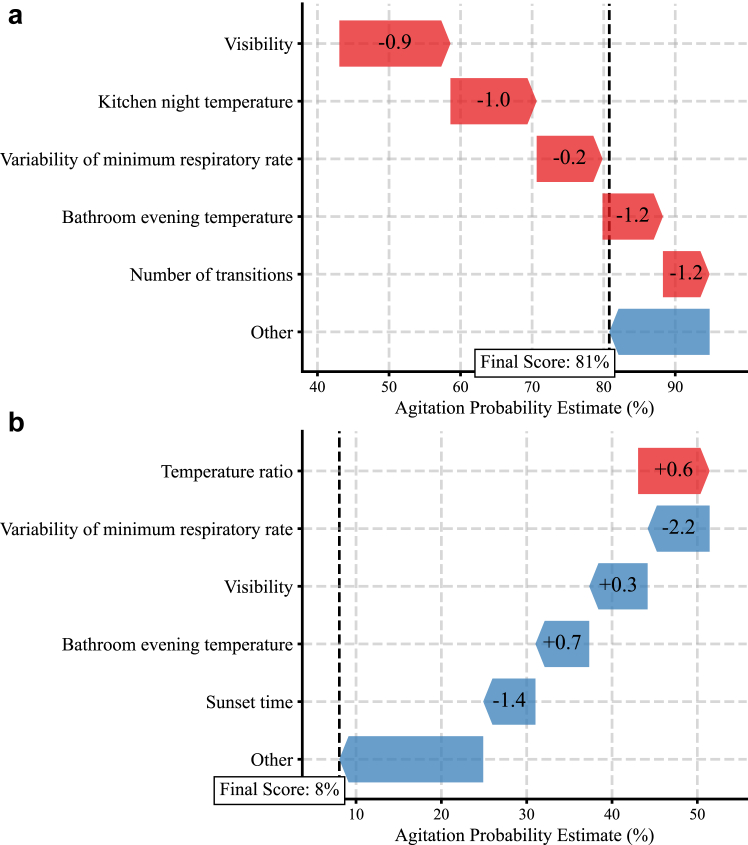
**Personalised investigation of modifiable features**. Examples from a week with presence of agitation (a) and a week without agitation (b) for PLwD A are shown using the SHapley Additive exPlanations (SHAP) framework. The colour of the arrow corresponds to the contribution: red contributes to agitation presence and blue contributes to agitation absence. Positive SHAP values contributed to positive predictions (agitated), while negative SHAP values contributed to negative predictions (non-agitated). The size of the arrow represents the absolute SHAP value, indicating the magnitude of each feature's contribution. The number within the arrow corresponds to the normalised feature value.

The SHAP framework allows further analysis of the features contributing to the model decision-making for an individual week. Fig. 3 shows two examples of one week with presence of agitation and one week without agitation from the same PLwD. Such insights can identify personalised interventions.

**Fig. 4:**
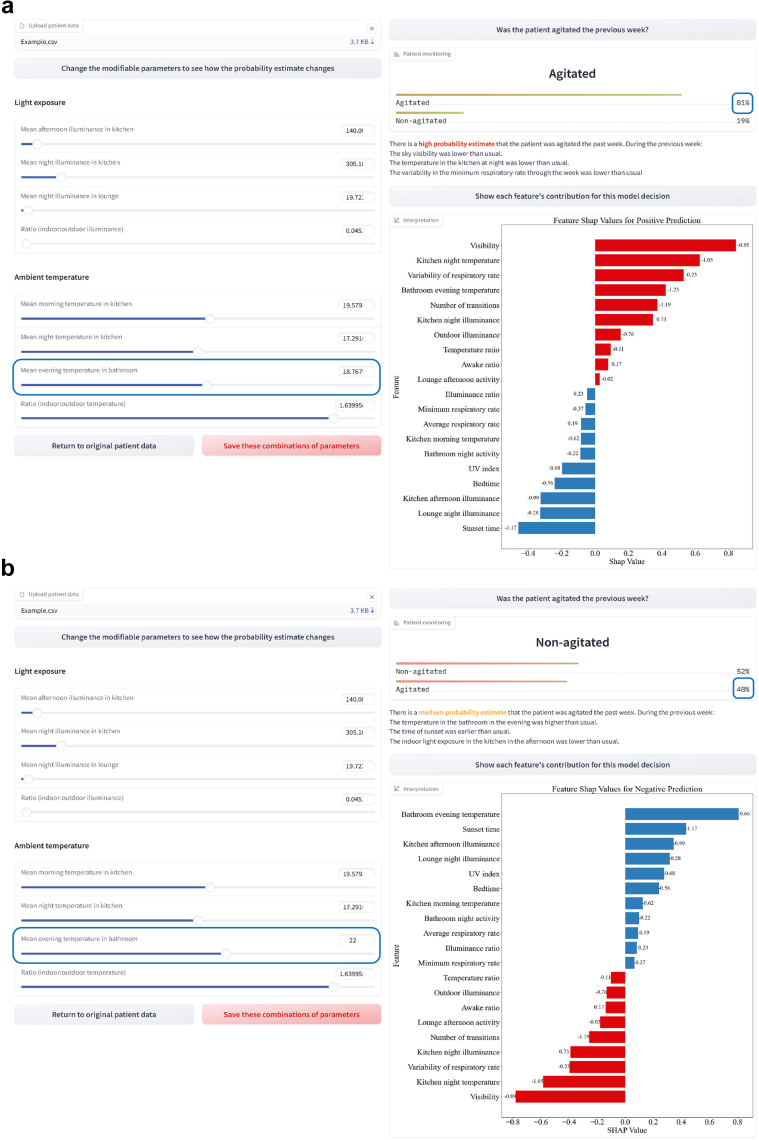
***In-silico* experiment**: Adjusting Temperature via an Interactive Interface. The interactive interface is shown, which accepts the input data as a CSV file. The tool provides sliding bars for the modifiable features and presents the associated probability estimates, and a feature importance plot using SHapley Additive exPlanations (SHAP) values. In the feature importance plot, red bars correspond to features that contributed to the model's decision-making towards agitation and blue bars correspond to features that contribute towards absence of agitation. Each bar is annotated with the corresponding normalised feature value. The user can save the combinations of modifications they have made to the modifiable parameters. a. Anonymised data from a participant. b. The results after modifying one of the parameters, evening indoor bathroom temperature. An online version with a synthetic patient data generator is hosted on huggingface (see https://huggingface.co/spaces/marirena/AgitationMonitoring).

To improve the clinical applicability of such analyses, we designed an interactive tool that allows clinicians to investigate the effect of the features contributing to the model's decision at an individual level. The tool provides a visualisation of the individual event and each feature's contribution to the probability estimates outputted by the model (Fig. 4).

We used this interactive interface to perform *in-silico* experiments by exploring the effect of non-pharmacological interventions on the estimated probability estimates of agitation. Through sliding bars, modifications of features can be simulated. The set of modifiable features investigated included 4 features on room temperature and 4 features on light exposure. By changing the values of these features, the model is re-run with the updated data, leading to a new agitation probability estimate. Exemplary, we demonstrated that by only modifying the evening indoor temperature in the bathroom from 18.77 to 22, the agitation probability estimate for a participant who was initially identified as having a high probability estimate of having experienced agitation (Red group) changed from 81% to 48%, shifting them to medium probability estimate (Amber) (Fig. 4). Similarly, for a PLwD whose afternoon kitchen illuminance was identified as an important factor (Figure S9), reducing the illuminance from 257.7 to 100 resulted in a probability estimate decrease from 77% to 23% (Figure S10) shifting the traffic-light category from medium (Amber) to low (Green). Such *in-silico* experiments enable clinicians to investigate personalised interventions.

## Discussion

We used a unique dataset from an in-home monitoring dementia care study to identify and investigate episodes of agitation in PLwD. A LightGBM classifier was employed to identify the presence of agitation over 8-day periods. Integration of feature importance analysis alongside a traffic-light system significantly increased the clinical utility of this ML framework. The development of an interactive online platform facilitated a comprehensive *in-silico* examination of non-pharmacological interventions.

The retrospective analysis of weekly agitation episodes, a novel approach within our study, provides opportunities to explore the underlying factors related to agitation. When comparing our model performance with existing literature, it is crucial to consider that our aim differed from that of previous models, which focused on detecting the onset of agitation. Spasojevic et al.^13^ achieved a higher ROC-AUC score (82.00%) than ours (77.63%) when incorporating a larger feature set (37 vs. our 20). However, with a comparable subset of features (18 vs. our 20), our model demonstrated similar ROC-AUC performance to theirs (77.4% vs. our 77.63%). Khan et al. using sequential deep modelling achieved higher ROC-AUC than our model (81.2% vs. our 77.63%).^36^ However, both studies were limited by a smaller participant sample (n = 20) compared to ours (n = 63) and provided limited model interpretability. Compared to our previous study by Palermo et al.,^14^ our proposed model achieved lower sensitivity (71.36% vs. 79.78%) but higher precision and F1-score. This suggests a decrease in false alerts in our current model, increasing its clinical applicability. Our proposed model balanced both high sensitivity and specificity, ensuring sensitive monitoring, while reducing unnecessary alerts (Table 2, Figure S8).

Addressing fairness and bias, our analysis indicates an absence of bias towards specific groups within our cohort (see Table S8, Table S9, Figure S3), likely attributable to the personalised pre-processing employed. A large body of ML research currently focuses on developing accurate methods for personalised pre-processing.^37^ Here, we scaled each feature to the baseline of each individual participant to accommodate underrepresented groups within our dataset. By considering individual baselines, we aimed to ensure that normal variations between groups were appropriately accounted for, without being conflated as indicators of agitation. This is the first study on ML applications for agitation that investigates model bias and model fairness. By doing so, we offer objective insights into this automated weekly monitoring process.

Through rigorous feature analysis, our model's interpretability and, by extension, its clinical utility have been significantly enhanced. Previous ML applications for agitation lacked insights into agitation-related factors due to their more complex models. By incorporating a wide range of longitudinal data, including sleep, activity, and importantly, indoor and outdoor environmental factors, which have not been extensively investigated, our model revealed significant agitation indicators that are not captured in routine clinical practice. Agitation periods in our study exhibited a notably higher awake ratio during sleep, suggesting heightened alertness and decreased sleep quality. Additionally, we observed a significantly lower minimum nocturnal respiratory rate, potentially indicating nocturnal breathing cessations (Fig. 2, Table S14). Extreme levels of light exposure, both high and low, were prevalent during agitation periods, alongside a significantly low illuminance ratio indicative of poor light quality (Fig. 2, Table S21). Low temperatures in the bathroom were also present during agitation periods (Fig. 2, Table S20). Our findings are consistent with previous clinical studies suggesting a link between sleep-disordered breathing and frequent awakenings with agitation.^38^^,^^39^ Similarly, our observations align with prior research reporting associations of extremely low illuminance levels at night with falls and hallucinations in older adults, potentially exacerbating agitation symptoms.^40^^,^^41^ Our finding of reduced light quality influencing agitation corresponds to an observational study reporting lower illuminance ratios in areas where PLwD tend to become agitated.^42^ Our observation that significantly higher illuminance values are associated with agitation is supported by studies indicating that high illuminance can cause discomfort due to age-related eye sensitivity.^43^ However, other studies that administered bright light therapy (BLT) to PLwD have shown mixed effects of high illuminance on agitation.^44^, ^45^, ^46^ Notably, illuminance levels during agitation weeks in our study remained relatively dim, with the highest average being 455.18 lux (Table S15), contrasting with BLT protocols that often exceed 1000 lux.^44^, ^45^, ^46^ It is also possible that the higher illuminance values recorded at night in the kitchen and lounge areas resulted from the patient's wandering throughout the house due to agitation. Our study showed that lower indoor temperatures were correlated with agitation, which relates to a previous finding where a deviation from an optimal temperature of 22.6 °C increased agitation behaviours.^47^ Overall, our model identified several known sleep, illuminance and ambient temperature features associated with agitation, including nighttime disturbances,^39^ sleep-disordered breathing,^38^ poor light quality^42^ and low ambient temperature.^47^ We further identified features that have not been previously associated with agitation in the literature, including extreme levels of outdoor visibility, pronounced differences between indoor and outdoor temperatures, as well as room and time-period specific illuminance and temperature measures, as illustrated in Fig. 2.

Our interpretable ML framework enables the exploration of non-pharmacological strategies to mitigate agitation and improve the quality of life for PLwD. Such strategies include the diagnosis and treatment of coexisting sleep disorders, particularly disordered breathing. Additionally, minimising nighttime disturbances is vital for alleviating carer distress, thereby supporting patients to remain at home and avoid institutionalisation.^48^ Our findings, along with current literature, suggest that modifiable parameters such as light exposure^49^ and temperature^47^ can be tailored for each PLwD to potentially reduce the recurrence of agitation episodes. Specifically, relying on natural light or adjusting artificial lighting to mimic natural light could improve light quality and alleviate agitation symptoms.^49^ Furthermore, maintaining indoor temperatures within a moderate range^47^ and avoiding significant fluctuations could enhance comfort and help reduce agitation episodes. Interventions targeting lighting levels in kitchen and lounge areas (Fig. 2) and adjustments to bathroom and kitchen temperature could prove beneficial in our cohort (Fig. 4).

This study highlighted ways to increase the clinical value of ML models through the automated, personalised weekly monitoring of agitation episodes, the inclusion of a traffic-light system and the development of an interactive interface, facilitating the exploration of personalised interventions.

Our model provides an alternative approach to the monitoring process that requires carers and patients to report agitation episodes. This method necessitates regular contact with patients and caregivers, which can be challenging in the long term. To make the monitoring process more effective while minimising disruptions, our model enables the clinical team to focus on contacting only those patients for whom an alert has been raised rather than reaching out to all patients each week. By employing an agitation monitoring model in real-world settings, we could enhance the detection of missed agitation instances, ultimately improving patient care and contributing to the development of more precise definitions for agitation episodes.

Identification of categories with regards to agitation probability estimates through a traffic-light system enables the effective management of urgent alerts (Red group), while minimising false alerts. The traffic-light system represents a comprehensive approach beyond binary predictions, a method not previously explored in other agitation monitoring models. This approach aligns with the NICE guidelines for monitoring serious diseases, highlighting its potential applicability in clinical practice.^50^ The traffic-light system has also been integrated by *Capstick* et al., for calculating the risk of urinary tract infections in PLwD, further demonstrating its broader relevance and clinical utility.^51^

The development of the first -to our knowledge-interactive tool for personalised intervention experimentation for agitation in dementia represents a significant advancement for dementia care. It aligns with current literature advocating for person-centred approaches to managing agitation in dementia.^22^ By utilising this tool, clinicians can offer practical instructions to PLwD and study partners for managing agitation.

The findings of this study are subject to limitations. Firstly, data quality may be compromised as participants reside with study partners (Table 1), potentially leading to the partners’ behaviour confounding the collected data. Future studies should use participant-specific data collection methods and investigate the role of study partners in assessing and managing agitation in PLwD. Secondly, the investigation of the potential effect of light exposure on behaviour is limited by only including illuminance data. Additional parameters such as spectral irradiance, bandwidth, and position of individuals relative to the light source have also been reported to influence behaviour.^52^ Thirdly, due to the uncontrolled environment, other external parameters that are not captured by our data collection could be affecting the behaviour.

Despite the model's fairness towards specific demographics within our cohort (Figure S3), it is crucial to acknowledge the lack of diversity in our dataset (Table 1). Addressing this limitation in future studies by recruiting participants from diverse demographic groups would not only ensure the fairness and generalisability of our framework across different populations but also allow for the external evaluation of our model. It should be noted that while we conducted feature selection, employed regularisation techniques and utilised grouped evaluation strategies to minimise overfitting, more data collection and further studies are required to validate generalisation of the proposed approach to larger cohorts and external data.

Further limitations arise from the use of a weekly assessment approach. The labels derived from weekly reports might be inaccurate due to subjective and incomplete recollections of events. Although the clinical research staff have received appropriate training to standardise the process, and we attempted to address labelling inaccuracies through a second review of the notes, the subjective nature of the assessments and inherent variability among different staff members may still introduce discrepancies. Nonetheless, our results demonstrate that employing ML models carefully and analysing longitudinal patterns allowed us to establish a reliable measure for identifying agitation patterns and episodes over weekly periods, that can serve as a valuable alternative to more subjective clinical assessments.

A further remark should be made on implied causality. Given our weekly approach to agitation detection, factors leading to agitation, alongside agitation behaviours, are included in the data. This could potentially render the identified markers epiphenomena rather than indicators of causality. To establish a causal relationship between environmental and sleep features, and agitation, controlled clinical intervention studies are needed. Our interface facilitates the identification of potential interventions to investigate.

Our next research phase involves conducting a clinical study to implement non-pharmaceutical interventions for agitation within our cohort, leveraging the developed model and tool. By employing this clinical decision-making support tool, we aim to assess its efficacy and clinical utility, while examining the effectiveness of the identified personalised interventions in managing agitation in dementia over the long term.

## Contributors

All authors have read and approved the final version of the manuscript.

MB, AC, and PB have accessed and verified the underlying data.

MB: Conceptualisation, Methodology, Software, Formal analysis, Investigation, Data Processing, Visualisation, Writing-Original Draft, Review and Editing; AKS: Methodology, Writing-Original Draft, Review and Editing; NFL: Supervision, Writing-Original Draft, Review and Editing; CW: Data collection, Writing-Review and Editing; AC: Provided visualisation package AVT, Writing-Review and Editing; CS, SK: Writing-Review and Editing; RN: Clinical Study Lead, Data Collection, Conceptualisation, Writing-Review and Editing, Funding Acquisition, Project Administration, Resources; PB: Conceptualisation, Methodology, Writing-Original Draft, Review and Editing, Supervision, Funding Acquisition, Project administration, Resources.

## Data sharing statement

Unidentified patient data can become available from the corresponding author upon reasonable request. The trained model is available at: https://huggingface.co/spaces/marirena/AgitationMonitoring/tree/main.

## Declaration of interests

All authors declare no competing interests.
